# Needs, Acceptability, and Functional Requirements of Gamified Cardiopulmonary Resuscitation Training Kits Among Children and Their Parents in China: Cross-Sectional Qualitative Study

**DOI:** 10.2196/88145

**Published:** 2026-08-07

**Authors:** Pan Wang, Zeya Shi, Furong Xu, Ying Huang, Yu Tian

**Affiliations:** 1 Emergency Department of Hunan Provincial People's Hospital, The First Affiliated Hospital of Hunan Normal University, Changsha, Hunan Province, China; 2 On-site Rescue Research Center of Hunan Provincial Geriatric Institute, Changsha, Hunan Province, China; 3 Hunan Prevention and Treatment Institute for Occupational Diseases, Affiliated Prevention and Treatment Institute for Occupational Diseases of University of South China, No. 162 Xinjian West Road, Chuangxin Bldg, 5th Fl, Changsha, Hunan Province, 410005, China, 86 13755091558; 4 School of Basic Medical Sciences, Hunan Normal University, Changsha, Hunan Province, China

**Keywords:** needs, acceptability, gamified, CPR training kits, children, parents

## Abstract

**Background:**

Out-of-hospital cardiac arrest (OHCA) is a global health issue with low survival rates, for which bystander cardiopulmonary resuscitation (CPR) is crucial. In China, bystander CPR rates and public proficiency remain low. Children—an important future first-responder group—also lack adequate training. Gamification and virtual reality (VR) technologies show potential in enhancing training effectiveness, but research on the needs, acceptability, and functional requirements of gamified CPR training kits from children’s and parents’ dual perspectives is insufficient, providing a realistic basis for this study.

**Objective:**

This study aimed to investigate children’s and parents’ needs, acceptability, and functional requirements for the development of gamified CPR training kits.

**Methods:**

A cross-sectional qualitative design was adopted. Inclusion criteria were children aged 6 to 12 years, their parents, adequate communication skills, and voluntary informed consent, with purposive sampling to recruit 13 parent-child dyads from a primary school in Changsha, Hunan Province. The participants included children aged 6 to 12 years (average age 9.50, SD 1.50 y, 6 boys and 7 girls) and their parents (average age 38.50, SD 2.50 y, 6 female and 7 male individuals). Semistructured interviews were conducted separately for children and parents, with audio recording and verbatim transcription. The NVivo 12.0 (QSR International Pty Ltd) software was used for thematic coding and analysis, and dual independent coding (by 2 researchers) was performed to ensure rigor, with discrepancies resolved through team discussion. Thematic saturation, defined as codebook stabilization and no new themes across 3 consecutive interviews, was achieved after 13 dyads. The study followed the COREQ (Consolidated Criteria for Reporting Qualitative Research) guidelines, and all procedures were approved by the ethics committee.

**Results:**

Four main themes and 9 subthemes emerged from the data: (1) children’s approaches to acquiring CPR-related knowledge and skills (through school-organized first aid activities, family members, or social media platforms), (2) attitudes toward gamified training kits (children’s receptiveness and parental preferences regarding such tools), (3) expectations for gamified CPR training kits (child-friendly design, effective skill training, personalized feedback, and scientifically grounded content), and (4) challenges in gamified science education (ensuring product safety and professionalism and contextualizing game scenarios to everyday life).

**Conclusions:**

As children are an important reserve force for future first responders, cultivating their first aid awareness is crucial for improving public emergency response capabilities. Effective gamified CPR training kit development requires integrating children’s and parents’ perceptions to balance entertainment, education, and practicality. This study innovatively explores dual user needs, differing from prior research focused on training outcomes rather than predevelopment requirements. It addresses gaps in pediatric first aid tool design and provides evidence-based references for the development of child-friendly, professional, gamified training resources—laying a foundation for gradually enhancing CPR literacy and emergency response awareness among children in China.

## Introduction

Cardiac arrest represents a critical public health emergency, with out-of-hospital cardiac arrest (OHCA) survival rates remaining low worldwide [1,2]. In China, the prospective BASIC-OHCA registry reported a crude emergency medical services–assessed OHCA incidence of 95.7 per 100,000 population in 2020, and the overall survival rate at hospital discharge or 30 days was only 1.2% [3]. This survival figure is substantially lower than the 10% to 16% reported in high-income settings, such as the United States [4]. The provision of immediate and high-quality cardiopulmonary resuscitation (CPR) by bystanders is the single most crucial factor in improving survival outcomes [5,6]. In China, however, the prevalence of bystander CPR training remains critically insufficient. A 2023 survey of Chinese middle school students found that only 9.87% had received CPR training [7], and a 2024 study of university freshmen reported that 82.61% cited a lack of first aid knowledge as the main barrier to performing CPR [8].

Global resuscitation bodies, including the International Liaison Committee on Resuscitation (ILCOR) and the European Resuscitation Council (ERC), have promoted universal first aid education. ILCOR launched the World Restart a Heart initiative, while the ERC’s 2015 Kids Save Lives campaign advocates formal school-based CPR training [9]. Many high-income countries (eg, the United States, Norway, and Japan) have mandated CPR training in primary and secondary school curricula through legislation, a policy that has significantly improved bystander CPR rates [10]. In China, however, this skills deficit is particularly pronounced among children—potential future first responders—a group that currently receives minimal effective training [11]. School-age children aged 6 to 12 years have sufficient cognitive ability for basic CPR [10], which corresponds to the primary school stage in China—a critical period for health education. Early first aid education fosters long-term response capabilities, and skills acquired can be retained throughout life. Moreover, children can share first aid knowledge with families and friends, thereby contributing to broader public awareness.

Current approaches to CPR training for children face several critical challenges, including a shortage of instructors, monotonous training formats that are insufficient to maintain engagement, and a lack of intrinsic motivators [12,13]. Moreover, existing training models and equipment may not be optimally aligned with children’s developmental characteristics (eg, shorter attention spans and a need for play-based learning) [14]. Consequently, conventional methods may fail to sustain children’s engagement, a key factor for long-term retention.

The 2020 AHA Guidelines for Cardiopulmonary Resuscitation and Emergency Cardiovascular Care suggest that gamified learning may be considered for CPR training among lay rescuers [15]. Gamification uses elements such as points, badges, and leaderboards to boost learners’ engagement and self-efficacy and has been shown to improve skill mastery, knowledge levels, and long-term retention [16,17]. A 2024 systematic review and meta-analysis of randomized controlled trials, published in *JMIR Serious Games*, concluded that serious games are comparable to traditional training methods in terms of theoretical knowledge, skill assessment, compression depth, and chest compression rate [18]. Recent interventional studies have further demonstrated that game-based approaches—including mobile apps, board games, and virtual reality (VR)–based scenarios—increase motivation and short-term skill retention in children aged 6 to 12 years [19,20]. VR and serious games further enhance this approach by providing immersive, safe environments for repetitive practice and by delivering immediate performance feedback [21-24].

Nevertheless, research exploring user-centered perspectives on gamified CPR training kits for children remains scarce. Existing studies have largely focused on short-term learning outcomes rather than user-centered acceptability and functional requirements [18,25]. The acceptability of such tools among children remains unclear. Likewise, their functional requirements from the perspectives of both children and their parents are largely unknown. To address this gap, this study aims to explore the needs, acceptability, and functional requirements for gamified CPR training kits among children aged 6 to 12 years and their parents in China, using a qualitative interview design. In this study, “needs” refers to participants’ perceived necessity for CPR training and their desired functional features (eg, personalization, real-time feedback, and social learning). The findings will identify key functional requirements from the dual perspectives of children and their parents, thereby providing targeted design references for the development of child-friendly, professional, gamified CPR training kits.

## Methods

### Research Design Overview

This study used a cross-sectional qualitative design using semistructured interviews. The study adopted a pragmatic epistemological stance, prioritizing the generation of actionable knowledge for intervention design while acknowledging that participants’ accounts reflect their situated realities. The central focus of this study was participants’ acceptability of gamified CPR training kits, operationally defined as their willingness to use and recommend the kits. The interviews also explored how specific kit features—namely interactive design, operational difficulty, and content relevance—are related to participants’ acceptability. The interview method was selected for its ability to elicit rich, detailed narratives and clarify complex perceptions regarding the acceptability and functional requirements of gamified CPR training kits. Furthermore, interviewing child-parent dyads was essential to capture the interdependent dynamics and potential consensus or divergence in opinions within the family unit, which individual interviews might overlook.

### Study Participants or Data Sources

The final cohort therefore consisted of 13 child-parent dyads (children: C1-C13; parents: P1-P13). To create a comfortable environment for children to express their views openly, interviews were conducted separately for the children and their parents. The mean age of the children was 9.50 (SD 1.50; range 6‐12) years, and the mean age of the parents was 38.50 (SD 2.50; range 35‐45) years. Children’s age and gender, as well as parents’ educational background, were considered participant characteristics of analytical interest during purposive sampling and analysis. No refusals or dropouts occurred.

### Participant Recruitment

#### Overview

From August 2024 to January 2025, we recruited child-parent dyads from an elementary school in Changsha, Hunan Province, using purposive sampling to ensure diverse representation in terms of children’s age, gender, and parents’ educational background and occupation. The inclusion criteria were as follows: (1) children aged between 6 and 12 years, (2) parents of children within this age range, (3) adequate communication skills, and (4) voluntary participation with informed consent obtained. The exclusion criteria were as follows: (1) cognitive impairments or psychiatric disorders in either the child or parent and (2) inability to complete the interviews.

#### Sample Size and Saturation

The sample size was determined based on the principle of thematic saturation. We conducted interviews with an initial 7 dyads and performed a preliminary analysis. Recruitment and analysis continued iteratively until no new themes emerged from subsequent interviews; saturation was achieved after including 6 additional dyads. Saturation was defined as codebook stabilization with no new themes emerging across 3 consecutive interviews. This assessment was performed across all interviews combined (rather than separately for children and parents) owing to the interdependence within each dyad.

### Data Collection

The original English interview guide was translated into Mandarin Chinese by a native Chinese-speaking researcher. A second bilingual researcher independently back-translated the Chinese version into English, and the research team compared the back-translated version with the original version to ensure conceptual equivalence. The final Chinese version was pilot-tested with 2 child-parent dyads (not included in the final sample) to verify cultural and linguistic appropriateness.

September 2024 to January 2025 served as the data collection period. The interviews adhered to the following standardized procedure: (1) preparation: all interviews were conducted in a private, quiet room at the participating elementary school. The room was arranged to create a comfortable, nonthreatening atmosphere for children. The researcher arrived 10 to 30 minutes before each session to prepare the environment and audio recording equipment. To capture the distinct perspectives of both groups, children and their parents were interviewed separately. Pilot interviews with 2 child-parent dyads (not included in the final sample) were conducted prior to formal data collection to refine the interview guide. (2) Introduction and informed consent: upon arrival, the researcher provided a clear explanation of the study’s purpose, procedures, and data handling practices using child-friendly language. Written informed consent was obtained from all parents, and verbal assent was obtained from all children after confirming their understanding. Participants were informed that all obtained information would be used solely for research purposes and would remain confidential. Once consent for recording was obtained, the audio recorder was activated. (3) Discussion: the researcher introduced topics based on the prepared interview-guiding questions (see Textbox 1) and cultivated an environment where participants were encouraged to share their views freely. To ensure the children started with a common understanding, a brief (3‐5 min) age-appropriate introduction to CPR and when it is needed was provided using simple videos and slides. Simple verbal questions were then used to confirm each child’s basic comprehension before the formal interview began. During the interviews, the researcher maintained methodological neutrality by using bracketing techniques; using verbatim restatement for clarification; and posing neutral, reflective questions (eg, “Could you tell me more about that idea?”). When interviewing children, the researcher used simplified language and concrete examples to facilitate comprehension. Particular attention was paid to documenting nonverbal cues (eg, facial expressions, body language) and paralinguistic features (eg, tone of voice, enthusiasm). Each interview lasted approximately 15 to 40 minutes. (4) Conclusion: based on participants’ responses, the interviewer reviewed the main discussion points. Each participant was provided a chance to add additional comments. After the interview, participants were offered a small token of appreciation.

Textbox 1. **Semistructured interview guide for parents and children participating in a qualitative assessment of cardiopulmonary resuscitation (CPR) training needs in Changsha, China (August 2024-January 2025).**

1. For children: Could you describe how you typically learn about first aid, particularly CPR procedures?

2. For both children and parents:

- From your perspective, what essential functionalities should an ideal gamified CPR training toy incorporate to ensure it is both educational and engaging for children?
- What are your expectations regarding the training efficacy of gamified CPR kits compared to traditional methods? Please elaborate.
- What design elements (eg, narrative structure, reward systems, interface characteristics) would you prioritize when developing an age-appropriate gamified CPR training device?
- What potential challenges or barriers do you anticipate in implementing gamified CPR kits within training environments?

### Data Analysis

#### Overview

The interviews were transcribed verbatim by 2 independent researchers within 24 hours of each session. The transcripts included notations of notable nonverbal cues where appropriate. To ensure accuracy, we conducted member checking by returning the transcripts to participants to verify accuracy. The verified transcripts were then imported into NVivo 12.0 (QSR International Pty Ltd) to facilitate data management and coding. We analyzed the data using thematic analysis following the systematic 6-phase approach outlined by Braun and Clarke [26]. This analysis was conducted from a pragmatic epistemological perspective, focusing on identifying patterns and themes directly relevant to intervention design decisions for CPR training kits.

#### Codebook Stabilization

To ensure a stable codebook, we adopted an iterative team-based approach. After the first 4 interviews (2 child-parent dyads), 2 researchers independently performed line-by-line coding of the transcripts. They met to compare codes, discuss discrepancies, and merge or refine codes to form a preliminary codebook. This process was repeated after coding an additional 4 interviews (2 dyads), during which no new codes emerged and existing codes were considered sufficient to capture new data segments—indicating that codebook stabilization had been achieved. The final codebook was then applied to the remaining interviews.

#### Theme Development

Following codebook stabilization, 1 researcher coded the remaining transcripts. Through team discussion, we grouped similar codes into subthemes (eg, children’s receptiveness, parental preferences) and then combined these into main themes (eg, attitudes toward gamified training kits). A thematic map was drawn to visualize the theme structure. The resulting themes were checked against the original transcripts to confirm they accurately reflected participants’ views, and any disagreements were resolved through team consensus.

#### Researcher Reflexivity

The research team consisted of investigators with academic backgrounds in emergency medicine and medical education. All interviews were conducted by the first author (female), who had prior experience working with children. All researchers involved in interviewing and analysis had formal training in qualitative research methods and experience in conducting interviews with child and adolescent populations. The team acknowledged prior assumptions that gamified kits would be acceptable to children and that children would prefer game-like features. To minimize the influence of these preconceptions on data collection and analysis, we emphasized reflexivity through bracketing (eg, noting judgments or emotional responses during interviews) and maintained a reflexive journal throughout the study.

No prior relationship existed between researchers and participants before the study. Researchers maintained a neutral and supportive role during interviews, particularly with children, to minimize power imbalances. Clear boundaries were established regarding the educational and research nature of the interaction, and no identifiable personal relationships were established beyond the interview context.

#### Methodological Integrity

Trustworthiness was established through multiple strategies: (1) member checking, in which participants reviewed transcripts for accuracy; (2) thick description of participants and context to support transferability; (3) reflexive journaling to document researchers’ positions and potential influences; and (4) peer debriefing to challenge and refine interpretations.

### Ethical Considerations

Ethics approval was obtained from the Medical Ethics Committee of Hunan Provincial People’s Hospital (The First Affiliated Hospital of Hunan Normal University; approval number [2024]-236). Participant consent was obtained before interviews were conducted, following strict ethical procedures for underage research participants. This process involved first providing detailed study information to all parents or legal guardians, from whom written informed consent was secured. Subsequently, age-appropriate explanations were provided to each child, and verbal assent was obtained after confirming their understanding. This study involved original primary data collection; no secondary data or existing datasets were used in the analysis. Participants could withdraw at any time without any negative consequences. Furthermore, we implemented strict confidentiality measures to protect participant privacy. All personally identifiable information was anonymized during data transcription and was securely stored, with access restricted to authorized research team members. No financial compensation was provided to participants. A small souvenir (valued at approximately 10 CNY, equivalent to ~US $1.40 as of January 31, 2025) was offered as a token of appreciation after the completion of the interviews. No identifiable images, photographs, or videos were collected or included in this study. Therefore, no additional consent for visual materials was required, and no related files were uploaded. The study adheres to the COREQ (Consolidated Criteria for Reporting Qualitative Research) guidelines [27].

## Results

### Overview

**Figure 1.**
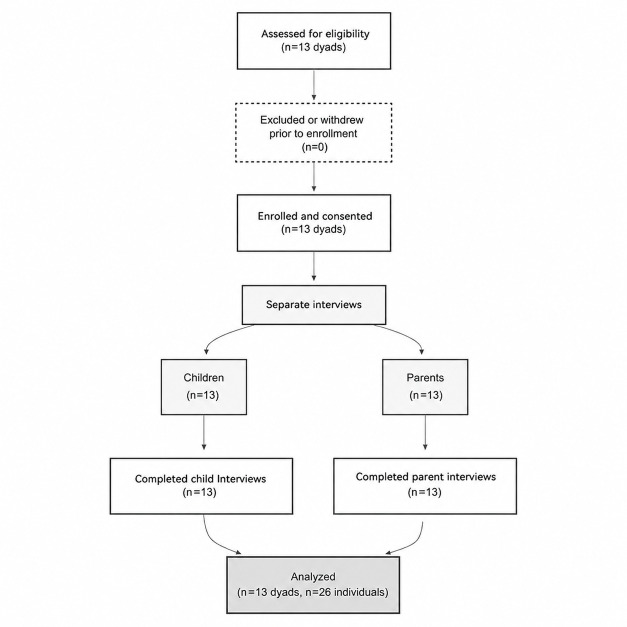
Participant flow diagram for a qualitative interview study on gamified cardiopulmonary resuscitation (CPR) training kits with parent-child dyads in Changsha, China (August 2024-January 2025).

**Figure 2.**
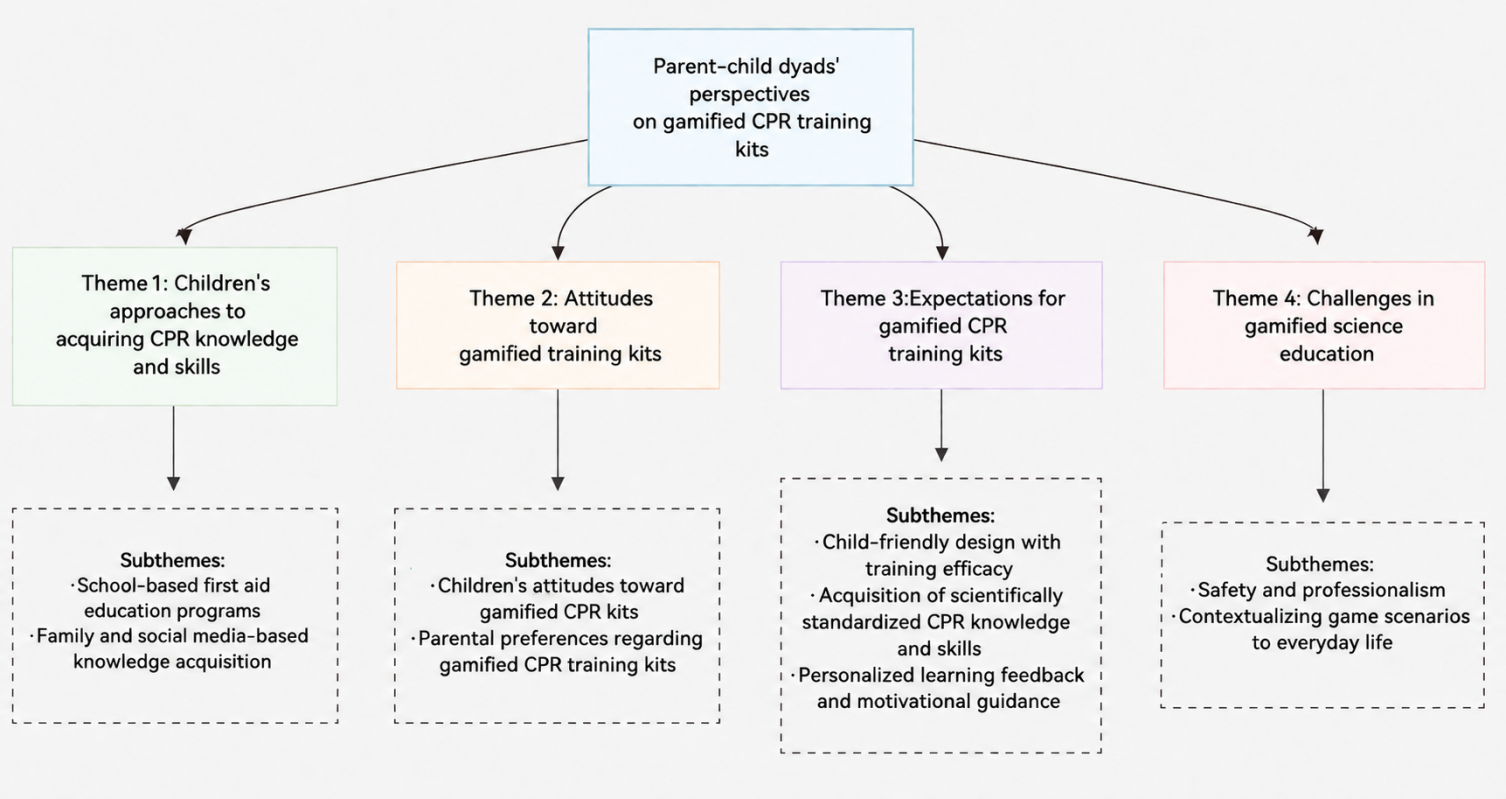
Thematic map of main themes and subthemes regarding gamified cardiopulmonary resuscitation (CPR) training kits identified from semistructured interviews with parent-child dyads in Changsha, China (August 2024-January 2025).

A total of 26 participants (n=13 children and n=13 parents; children’s average age: mean 9.50, SD 1.50 years; parents’ average age: mean 38.50, SD 2.50 years) were included in this study (Table 1). The participant recruitment process is shown in Figure 1. After an initial comprehensive review of the data, a total of 108 codes were developed, and 9 subthemes were identified. These subthemes were then further refined and 4 main themes emerged, including (1) children’s approaches to acquiring CPR-related knowledge and skills, (2) attitudes toward gamified training kits, (3) expectations for gamified CPR training kits, and (4) challenges in gamified science education (Table 2). Figure 2 presents a thematic map of these themes and subthemes.

**Table 1.** Demographic characteristics of parents and children in a qualitative interview study on cardiopulmonary resuscitation (CPR) training needs, Changsha, China (August 2024-January 2025).

Variable	Values	
	Parents (n=13)	Children (n=13)
Age (y), mean (SD)	38.50 (2.50)	9.50 (1.50)
Sex, n (%)		
Female	6 (46.15)	7 (53.85)
Male	7 (53.85)	6 (46.15)
Education level, n (%)		
High school or below	2 (15.38)	—^a^
Bachelor’s degree	6 (46.15)	—
Master’s degree or higher	5 (38.46)	—
Current grade, n (%)		
Grade 1‐2	—	2 (15.38)
Grade 3‐4	—	3 (23.08)
Grade 5‐6	—	8 (61.54)
Occupational background, n (%)		
Health care professional	2 (15.39)	—
Other occupations	9 (69.23)	—
Unemployed	2 (15.38)	—
Previous CPR training, n (%)		
Yes	5 (38.46)	—
No	8 (61.54)	—
Number of CPR training sessions, mean (SD)	—	0.69 (0.63)

a Not applicable.

**Table 2.** Main themes and subthemes regarding gamified cardiopulmonary resuscitation (CPR) training kits identified from semistructured interviews with parent-child dyads in Changsha, China (August 2024-January 2025).

Main themes	Subthemes
Children’s approaches to acquiring CPR-related knowledge and skills	School-based first aid education programs Family and social media-based knowledge acquisition
Attitudes toward gamified training kits	Children’s attitudes toward gamified CPR kits Parental preferences regarding gamified CPR training kits
Expectations for gamified CPR training kits	Child-friendly design with training efficacy Acquisition of scientifically standardized CPR knowledge and skills Personalized learning feedback and motivational guidance
Challenges in gamified science education	Safety and professionalism Contextualizing game scenarios to everyday life

### Modalities of CPR Knowledge Acquisition Among Children

#### School-Based First Aid Education Programs

Children predominantly acquired CPR knowledge through structured first aid initiatives conducted by professional medical teams within school settings. While recognizing these programs as engaging and credible, participants consistently highlighted limitations in the duration of sessions and insufficient opportunities for hands-on practice. Representative verbatim accounts are as follows:

*Healthcare professionals such as doctors and nurses conducted 45-minute CPR training sessions at our school, combining theoretical instruction with practical demonstrations. However, the practice time was insufficient for all participants to sufficiently practice the techniques.*

[C2]

*The hospital-organized first aid lectures effectively utilized clinical case scenarios to enhance knowledge retention. Nevertheless, the limited availability of manikins limited the extent of our practical engagement during the training.*

[C3]

*During weekly safety education assemblies, teachers covered basic first aid concepts such as emergency call procedures, though CPR skill practice was notably absent from these sessions.*

[C5]

#### Family and Social Media–Based Knowledge Acquisition

Our analysis identified 2 distinct yet limited pathways for CPR knowledge acquisition beyond school settings. First, family-mediated learning occurred exclusively among children whose relatives were health care professionals (2/13 participants), characterized by improvised training using household items (pillows, stuffed animals) to demonstrate proper technique. Second, all participants reported exposure to CPR content through social media platforms (Douyin or Xiaohongshu) and television, though they expressed uniform skepticism regarding technical accuracy. Notably, emotionally compelling survival narratives (eg, child-saving-mother scenarios) significantly enhanced the perceived importance of CPR skills despite recognized knowledge gaps. Representative accounts include the following:

- C1: describing physician-led home practice (“My mother demonstrates compression rhythm using pillows”)
- C6: recalling impactful media stories (“The child hero narrative made me value CPR”)
- C10: observing platform limitations (“Douyin tutorials seem daunting without guidance”)

These findings underscore both the potential and the limitations of informal CPR education channels in pediatric populations.

### Perspectives on Gamified CPR Training Kits

#### Children’s Attitudes Toward Gamified CPR Training Kits

Despite limited prior exposure to first aid educational games, participants expressed universal recognition of the potential value of gamified CPR training tools. Three key themes emerged regarding their anticipated benefits: (1) significant enthusiasm for interactive elements and game mechanics (C5: “A toy that teaches CPR step-by-step would be extremely meaningful”), (2) enhanced learning engagement (C11: “Learning CPR through games seems both fun and educational”), and (3) potential to mitigate traditional learning barriers (C2: “Medical knowledge seems abstract, but gamification might make CPR techniques more accessible and memorable”). Notably, children’s positive expectations centered on game design’s potential to transform complex medical procedures into engaging, achievable tasks—a perception especially prevalent among those who had previously struggled with conventional training methods. These findings suggest that while pediatric users lack experience with existing products, they possess a nuanced understanding of gamification’s educational potential in skill acquisition contexts.

#### Parental Preferences Regarding Gamified CPR Training Kits

Parental perspectives revealed 3 primary considerations in evaluating gamified CPR training tools: (1) product design—emphasis on age-appropriate esthetics and cost-effectiveness (P11: “We regularly purchase educational kits and would consider a reasonably priced CPR training product”), (2) educational value—concerns regarding the balance between engagement and substantive learning outcomes (P3: “While innovative, many gaming kits prioritize entertainment over education—effective CPR tools must maintain this balance”), and (3) safety assurance—universal priority placed on material safety and child-friendly features (P1: “Convenience matters, but safety certification is non-negotiable”). Notably, parents demonstrated critical discernment, advocating for kits that integrate playfulness with medical accuracy—a demand unmet by existing products. These findings underscore the need for rigorously designed interventions that address parental concerns while meeting children’s learning needs.

### Expectations for Gamified CPR Training Kits

#### Child-Friendly Design With Training Efficacy

Stakeholders proposed 6 evidence-based design principles to enhance engagement and educational outcomes in gamified CPR training tools: (1) developmentally appropriate interfaces incorporating vibrant colors and cartoon characters (P3: “Narrative scenarios like rescuing a collapsed teddy with stepwise CPR guidance”), (2) progressive challenge systems featuring level-based advancement (P1: “Tiered difficulty modes sustain engagement with complex content”), (3) multimodal feedback mechanisms that combine haptic and auditory cues (P4: “Real-time corrective feedback such as vibration and verbal prompts ensure proper 30:2 compression-ventilation ratios”); (4) positive reinforcement systems with immediate verbal praise (P10: “Affirmative audio feedback enhances motivation”); (5) behavioral incentive structures including reward systems (C7: “Mission-based point accrual and leaderboards”), and (6) clinical fidelity maintenance ensuring medical accuracy (P4: “Must authentically simulate CPR mechanics beyond superficial gameplay”). These recommendations reflect a sophisticated synthesis of play pedagogy and resuscitation science, indicating that optimal designs must reconcile entertainment value with uncompromised training effectiveness through three core elements—(1) segmented skill-building modules; (2) authentic biomechanical feedback; and (3) intrinsic motivation systems—while maintaining American Heart Association (AHA)– or ERC-compliant performance standards.

#### Acquisition of Scientifically Standardized CPR Knowledge and Skills

Parents emphasized the critical need for gamified CPR training kits to integrate medically accurate content with engaging design features to overcome the limitations of traditional training methods. Three essential requirements emerged: (1) expert-validated content—parents strongly advocated for physician-reviewed instructional materials (P8: “The CPR content must be medically accurate, ideally verified by healthcare professionals, to ensure reliable learning”), (2) core skill mastery—a focus on teaching standardized techniques, including correct hand positioning, compression depth (5‐6 cm), rate (100‐120/min), and ventilation methods (P9: “Children must learn precisely where, how, and how fast to perform compressions, preferably through professional animated demonstrations”), and (3) retention optimization—strategies to address skill decay through repetitive practice (P12: “School training is easily forgotten without reinforcement—an engaging toy could enable regular practice”). Notably, parents demonstrated sophisticated understanding of AHA/ERC guidelines while recognizing the need for developmentally appropriate delivery methods. These findings highlight the necessity of balancing rigorous adherence to resuscitation science with child-centered pedagogical approaches in toy design.

#### Personalized Learning Feedback and Motivational Guidance

Analysis revealed two critical challenges in pediatric CPR skill acquisition: (1) difficulty maintaining technique accuracy and (2) low engagement during repetitive practice. Participants proposed four evidence-based solutions through gamified design: (1) real-time corrective feedback, as described by P3: “Children frequently forget proper positioning—the toy should provide immediate correction as an instructor would,” and P9: “Gentle corrective prompts for inadequate depth/rate (‘Press deeper’) are essential”; (2) adaptive difficulty systems, with P5 noting: “The toy should auto-adjust to children’s physical capabilities—different depth standards for preschoolers and older children”; (3) developmentally appropriate instruction, exemplified by P2: “Use child-friendly cues such as ‘Place hands in the center of teddy’s chest’ for positioning”; and (4) multimodal rhythm guidance, per P7: “Incorporate auditory/visual metronomes (eg, music or light cues) to pace compressions.” Parents particularly emphasized progression tracking systems to sustain motivation (P8: “Level-based advancement with visible progress metrics would maintain engagement”). These findings align with the Vygotsky zone of proximal development, suggesting that optimal kits should provide scaffolded, individualized feedback while adhering to AHA technique standards.

### Challenges in Gamified Science Education

#### Safety and Professionalism of Gamified Training Kits

Parents prioritized two fundamental requirements in gamified CPR training kits: (1) evidence-based educational value: over 80% (21/26) of the participants emphasized the primacy of medical accuracy over entertainment features (P8: “The core objective must be knowledge acquisition—engagement elements, while beneficial, should not compromise educational integrity”); and (2) child-safe design: 3 key safety concerns emerged as predominant: material safety (P13: “Battery safety standards must prevent thermal hazards”), ergonomic considerations (P4: “10-min segmented training modules to prevent fatigue”), and age-appropriate interfaces. Notably, stakeholders explicitly rejected purely entertainment-focused designs, advocating instead for pedagogically optimized tools designed to balance brief, focused sessions (≤10 min) with uncompromised resuscitation science fidelity. Collectively, these findings underscore the necessity for rigorous safety certifications (eg, CE/ISO markings) and medical expert validation throughout product development.

#### Contextualization of Game Scenarios in Daily Life

Both children and parents consistently favored realistic emergency scenarios over abstract game mechanics, emphasizing the imperative for contextual learning experiences. Participants specifically identified two critical needs: (1) authentic scenario simulation: the recreation of plausible emergency situations (C1: “Rather than complex rules, depict recognizable scenarios like assisting a collapsed classmate during running”) and (2) adaptive response systems: contextually adaptive feedback systems based on user actions (P13: “The toy should simulate real emergencies to enhance practical response capabilities”). These findings demonstrate a clear user demand for scenario-based training that facilitates the transfer of skills from game environments to real-world applications.

## Discussion

### Summary of Principal Findings

The main findings indicate that both children and their parents recognized the necessity of early CPR education and were receptive to gamified approaches, provided that safety and educational validity are assured. Both children and parents accepted gamification, but with different priorities: parents’ endorsement was conditional on safety and educational credibility, while children showed strong engagement with visual narratives and virtual scenarios. Despite these different priorities, both groups converged on a shared set of expectations for a training kit. Three core functional requirements were identified from the dual perspective: personalization, real-time interactive feedback, and social learning features. This study assessed acceptability—willingness to use and recommend—rather than effectiveness or usability.

### Interpretations and Comparisons

The demand for real-time, multimodal feedback can be effectively framed through the theory of self-efficacy [28]. Positive, performance-based feedback likely serves as a powerful source of mastery experience, enhancing a child’s belief in their capability to perform CPR correctly. For children who have never encountered medical procedures before, this external validation matters more than it would for adults—they cannot draw on past experience to judge their own performance, so they rely on what the feedback tells them in the moment [29]. This finding is consistent with previous research on game-based learning in health education, which shows that immediate feedback increases motivation and skill confidence [24,30]. Given children’s developing self-regulatory capacities, parents emphasized a balance between entertainment and educational substance, advocating for designs that prioritize skill acquisition [31]. This highlights that for this demographic, perceived usefulness is a prerequisite for parental acceptance, even within a highly gamified context [32].

A pivotal finding was children’s significantly greater engagement with visual narratives and virtual scenarios compared to conventional methods. The gamified approach may address 2 key barriers in CPR training among laypeople: the complexity of technical medical content and the anxiety associated with skill practice [33]. By simulating real-life emergencies in a safe, controllable, and repeatable game environment, this method may enhance a sense of mastery and self-efficacy, which could reduce initial apprehension and build operational confidence. Several parents in our study raised this exact concern that if the game design leans too far into entertainment and loses sight of medical accuracy, it could give children the wrong impression that cardiac arrest response is less serious than it actually is. Therefore, the design needs to find a middle ground—keeping children engaged while staying grounded in clinical reality—a balance that has been similarly emphasized in VR medical training studies [22,34].

Participants called for phased training objectives, which aligns with established findings that well-designed reward mechanisms bolster achievement motivation and goal attainment [35]. Competitive elements, as demonstrated by de Sena et al [36], were also acknowledged as effective drivers of program completion. That said, not all children in our study cared about rewards—a few said that it did not matter to them. This suggests that the effect of game mechanics may vary across individuals [37]. Consequently, these results suggest that optimal designs could implement scenario-based progressive learning, incorporate evidence-based motivational systems, and uphold rigorous medical accuracy across all gamified elements.

Parents in our interviews expressed high expectations regarding safety and educational value. Whether these expectations translate into actual adoption depends on the product’s reliability, educational worth, and cost [38]. At the school level, integration also hinges on curriculum flexibility and whether such tools can be situated within existing instructional routines [39]. At the policy level, experience from high-income countries suggests that incorporating CPR training into school curricula has relied on government support and resource allocation [10]; in China, relevant policy frameworks are still emerging, which presents both a challenge and an opportunity for developers and researchers to work with education authorities toward the systematic adoption of such tools [40]. Therefore, alongside the kit itself, user-friendly guidance materials and basic training protocols should also be developed to help teachers, children, and parents get started without friction, minimizing the risk of nonuse due to operational difficulties or inadequate preparation.

While previous research has measured CPR training outcomes in children [41-43], a notable gap exists in the co-design process that involves children users, which may have contributed to the low training coverage and implementation gaps reported in the literature [44]. This study fills this gap by systematically investigating the perceptions and requirements of both children and parents prior to development. Unlike prior studies that focused primarily on skill outcomes, our findings show that both children and parents view acceptability and user engagement as essential for a training tool to be effective. We also demonstrate that these perspectives can be elicited through qualitative methods before prototyping.

### Limitations

This study has several limitations. First, its qualitative nature provides rich insights into perceptions and attitudes but does not quantitatively measure actual CPR skill acquisition, retention, or behavioral outcomes in a real emergency. The expressed willingness to use such training kits may not directly translate into effective learning or action. Moreover, this study focused on acceptability rather than the physical realism of chest compression, which is essential for hands-only CPR training. The extent to which gamified tools alone can deliver the tactile feedback needed for skill mastery remains unknown based on this study. Second, the participant sample was drawn from a single elementary school in a specific sociocultural context (urban China), which further limits the generalizability of the findings to other regions, school settings, or cultural contexts with different attitudes toward play and education. In addition, the sample was limited to children aged 6 to 12 years, and the findings may not generalize to adolescents or other populations. Third, the small sample (13 dyads) from 1 school limits statistical generalizability, but qualitative research prioritizes detailed understanding over broad representation. Future multischool studies could test the transferability of our findings, and our volunteer sample may have introduced positivity bias.

### Conclusions

Gamified CPR training kits are acceptable to children and parents in China when designed with safety, medical accuracy, and engaging mechanics. This study moves beyond outcome-focused research by foregrounding the voices of end users in the co-design process. The identified design parameters—personalization, interactive feedback, social learning, and a balance between engagement and educational credibility—provide a road map for developing age-appropriate CPR training kits. Beyond the immediate design implications, our findings have broader significance. For developers, the requirements we identified can inform design standards, with particular attention to balancing entertainment and educational credibility. For educators and policymakers, our results support incorporating gamified CPR training into school health curricula, but only after kits have passed certified safety and medical accuracy checks. For researchers, this study demonstrates the value of capturing child and parent perspectives before prototyping; future research could move beyond acceptability to test actual learning outcomes through randomized controlled trials and examine how co-designed features influence long-term skill retention. These contributions advance a user-centered agenda for CPR education and gamified health tool design.

## Supplementary material

10.2196/88145 Checklist 1 COREQ checklist.

## Abbreviations

AHA: American Heart Association
COREQ: Consolidated Criteria for Reporting Qualitative Research
CPR: cardiopulmonary resuscitation
ERC: European Resuscitation Council
ILCOR: International Liaison Committee on Resuscitation
OHCA: out-of-hospital cardiac arrest
VR: virtual reality

## References

[1] Gräsner JT, Wnent J, Lefering R (2026). European registry of cardiac arrest study THREE (EuReCa- THREE)—EMS response time influence on outcome in Europe. Resuscitation.

[2] Wigginton JG, Agarwal S, Bartos JA (2025). Part 9: adult advanced life support: 2025 American Heart Association Guidelines for cardiopulmonary resuscitation and emergency cardiovascular care. Circulation.

[3] Zheng J, Lv C, Zheng W (2023). Incidence, process of care, and outcomes of out-of-hospital cardiac arrest in China: a prospective study of the BASIC-OHCA registry. Lancet Public Health.

[4] Tsao CW, Aday AW, Almarzooq ZI (2023). Heart disease and stroke statistics-2023 update: a report from the American Heart Association. Circulation.

[5] Soar J, Böttiger BW, Carli P (2025). European Resuscitation Council Guidelines 2025 adult advanced life support. Resuscitation.

[6] Leng Z, Li J, Tang K, Shi J (2026). Effect of bystander CPR on survival after out of hospital cardiac arrest: a systematic review and meta-analysis. Shock.

[7] Li Y, Xiong D, Xu L, Jin X (2023). Attitudes and willingness toward out-of-hospital CPR and AED: a questionnaire study among Chinese middle school students. Heliyon.

[8] Qin Z, Zheng S, Liu C (2024). The knowledge, training, and willingness of first year students in Xuzhou, China to perform bystander cardiopulmonary resuscitation: a cross-sectional study. Front Public Health.

[9] Böttiger BW, Bossaert LL, Castrén M (2016). Kids save lives—ERC position statement on school children education in CPR: “Hands that help—training children is training for life”. Resuscitation.

[10] Schroeder DC, Semeraro F, Greif R (2023). KIDS SAVE LIVES: basic life support education for schoolchildren: a narrative review and scientific statement from the International Liaison Committee on Resuscitation. Circulation.

[11] McGlinchey Ford M, Rogotzke CD, Bencik SL (2024). Teaching cardiopulmonary resuscitation to later elementary school students. Ann Emerg Med.

[12] Parisis C, Bouletis A, Chatzidimitriou K (2021). The impact of kids save lives program on knowledge, skills and attitude of Greek students. Final results from 2 years of implementation. Eur Heart J Acute Cardiovasc Care.

[13] Wingen S, Jeck J, Schroeder DC, Wingen-Heimann SM, Drost RMWA, Böttiger BW (2022). Facilitators and barriers for the implementation of resuscitation training programmes for schoolchildren: a systematic review. Eur J Anaesthesiol.

[14] Semeraro F, Imbriaco G, Del Giudice D (2024). Empowering the next generation: an innovative “Kids Save Lives” blended learning programme for schoolchildren training. Resuscitation.

[15] Panchal AR, Bartos JA, Cabañas JG (2020). Part 3: adult basic and advanced life support: 2020 American Heart Association Guidelines for cardiopulmonary resuscitation and emergency cardiovascular care. Circulation.

[16] Tung WS, Baker R, Toy K (2024). Gamification and serious games in orthopedic education: a systematic review. Cureus.

[17] Rodríguez-García A, Ruiz-García G, Navarro-Patón R, Mecías-Calvo M (2024). Attitudes and skills in basic life support after two types of training: traditional vs. gamification, of compulsory secondary education students: a simulation study. Pediatr Rep.

[18] Cheng P, Huang Y, Yang P (2024). The effects of serious games on cardiopulmonary resuscitation training and education: systematic review with meta-analysis of randomized controlled trials. JMIR Serious Games.

[19] Flato UAP, Flato A, Martins IBDT (2025). Enhancing equity in schoolchildren’s basic life support education in Brazil through serious games: cohort study. JMIR Serious Games.

[20] Flato UAP, Beffa Dos Santos EJ, Bispo Diaz T Martins I (2024). Interactive serious game to teach basic life support among schoolchildren in Brazil: design and rationale. JMIR Serious Games.

[21] Siqueira TV, Nascimento JDSG, Oliveira JLGD, Regino DDSG, Dalri MCB (2020). The use of serious games as an innovative educational strategy for learning cardiopulmonary resuscitation: an integrative review [Article in English, Portuguese]. Rev Gaucha Enferm.

[22] Sun R, Wang Y, Wu Q (2024). Effectiveness of virtual and augmented reality for cardiopulmonary resuscitation training: a systematic review and meta-analysis. BMC Med Educ.

[23] Chen Z, Chen Y, Zhang L, He Z (2026). Using serious games for cardiopulmonary resuscitation training: a meta-analysis and systematic review. Front Public Health.

[24] Wingen S, Großfeld N, Adams NB (2024). Do laypersons need app-linked real-time feedback devices for effective resuscitation?—Results of a prospective, randomised simulation trial. Resusc Plus.

[25] Boggs S, McNally JD, O’Hearn K (2024). Teaching high quality paediatric basic life support to laypeople: the development and evaluation of a virtual simulation game. Resusc Plus.

[26] Braun V, Clarke V (2006). Using thematic analysis in psychology. Qual Res Psychol.

[27] Tong A, Sainsbury P, Craig J (2007). Consolidated criteria for reporting qualitative research (COREQ): a 32-item checklist for interviews and focus groups. Int J Qual Health Care.

[28] Wang X, Mao M, Qian D (2025). Effectiveness of the flipped classroom on medical undergraduates’ cardiopulmonary resuscitation training in large class through self-efficacy: a randomized quasi-experimental study. J Med Educ Curric Dev.

[29] Bennett-Pierre G, Chernuta T, Altamimi R, Gunderson EA (2024). Effects of praise and “easy” feedback on children’s persistence and self-evaluations. J Exp Child Psychol.

[30] Wang SA, Su CP, Fan HY, Hou WH, Chen YC (2020). Effects of real-time feedback on cardiopulmonary resuscitation quality on outcomes in adult patients with cardiac arrest: a systematic review and meta-analysis. Resuscitation.

[31] Keller AS, Sydnor VJ, Pines A, Fair DA, Bassett DS, Satterthwaite TD (2023). Hierarchical functional system development supports executive function. Trends Cogn Sci.

[32] Allan KS, Mammarella B, Visanji M (2023). Methods to teach schoolchildren how to perform and retain cardiopulmonary resuscitation (CPR) skills: a systematic review and meta-analysis. Resusc Plus.

[33] Maher CA, Olds T, Vandelanotte C (2022). Gamification in a physical activity app: what gamification features are being used, by whom, and does it make a difference?. Games Health J.

[34] Alcázar Artero PM, Pardo Rios M, Greif R (2023). Efficiency of virtual reality for cardiopulmonary resuscitation training of adult laypersons: a systematic review. Medicine (Baltimore).

[35] Li S, Liu G, Qiao L (2026). Design requirements for gamified exercise apps for adults with prehypertension based on the Octalysis Framework and Self-Determination Theory: qualitative interview study. JMIR Serious Games.

[36] de Sena DP, Fabrício DD, da Silva VD, Bodanese LC, Franco AR (2019). Comparative evaluation of video-based on-line course versus serious game for training medical students in cardiopulmonary resuscitation: a randomised trial. PLoS One.

[37] Yao J, Song D, Xiao T, Zhao J (2025). Smartwatch-based tailored gamification and user modeling for motivating physical exercise: experimental study with the maximum difference scaling segmentation method. JMIR Serious Games.

[38] Reddy S (2024). Generative AI in healthcare: an implementation science informed translational path on application, integration and governance. Implement Sci.

[39] Da’Costa A, Teke J, Origbo JE, Osonuga A, Egbon E, Olawade DB (2025). AI-driven triage in emergency departments: a review of benefits, challenges, and future directions. Int J Med Inform.

[40] Li S, Qin C, Zhang H (2024). Survival after out-of-hospital cardiac arrest before and after legislation for bystander CPR. JAMA Netw Open.

[41] Otero-Agra M, Barcala-Furelos R, Besada-Saavedra I, Peixoto-Pino L, Martínez-Isasi S, Rodríguez-Núñez A (2019). Let the kids play: gamification as a CPR training methodology in secondary school students. A quasi-experimental manikin simulation study. Emerg Med J.

[42] Pitz Durič N, Borovnik Lesjak V, Strnad M (2024). Comparison of effectiveness of two different practical approaches to teaching basic life support and use of an automated external defibrillator in primary school children. Medicina (Kaunas).

[43] de Oliveira Martins LF, Melo AJB, Reis DB, Alves MG (2023). Evaluation of the efficiency of the different methods of teaching cardiopulmonary resuscitation to children and adolescents: integrative review. HSJ.

[44] Tamirisa K, Patel H, Karim S, Mehta NK (2023). Current landscape in US schools for bystander CPR training and AED requirements. J Interv Card Electrophysiol.

